# Calcitonin-secreting neuroendocrine neoplasms of the lung: a systematic review and narrative synthesis

**DOI:** 10.1530/EC-21-0071

**Published:** 2021-03-24

**Authors:** David C Llewellyn, Rajaventhan Srirajaskanthan, Royce P Vincent, Catherine Guy, Eftychia E Drakou, Simon J B Aylwin, Ashley B Grossman, John K Ramage, Georgios K Dimitriadis

**Affiliations:** 1 Department of Endocrinology ASO/EASO COM, King’s College Hospital NHS Foundation Trust, Denmark Hill, London, UK; 2 Neuroendocrine Tumour Unit, Kings Health Partners ENETS Centre of Excellence, Denmark Hill, London, UK; 3 Faculty of Life Sciences and Medicine, Kings College London, London, UK; 4 Department of Clinical Biochemistry, King’s College Hospital NHS Foundation Trust, Denmark Hill, London, UK; 5 Department of Cellular Pathology, Royal Sussex County Hospital, Eastern Road, Brighton, UK; 6 Department of Clinical Oncology, Guy’s Cancer Centre – Guy’s and St Thomas’ NHS Foundation Trust, Great Maze Pond, London, UK; 7 Oxford Centre for Diabetes, Endocrinology and Metabolism, University of Oxford, Oxford, UK; 8 Barts and the London School of Medicine, Centre for Endocrinology, William Harvey Institute, London, UK; 9 Neuroendocrine Tumour Unit, Royal Free Hospital, London, UK; 10 Faculty of Life Sciences and Medicine, School of Life Course Sciences, Obesity Immunometabolism and Diabetes Group, King’s College London, London, UK

**Keywords:** calcitonin, ectopic, paraneoplastic, neuroendocrine neoplasm, lung, systematic review

## Abstract

Calcitonin-secreting neuroendocrine neoplasms of the lung are rare, with few cases reported in the literature. Differentiating between medullary thyroid carcinoma and an ectopic source of calcitonin secretion can represent a complex diagnostic conundrum for managing physicians, with cases of unnecessary thyroidectomy reported in the literature. This manuscript reports a case of ectopic hypercalcitonaemia from a metastatic neuroendocrine neoplasm of the lung with concurrent thyroid pathology and summarises the results of a systematic review of the literature. Medical Literature Analysis and Retrieval System Online, Excerpta Medica, Cochrane Central Register of Controlled Trials, ClinicalTrials.gov and SCOPUS databases were systematically and critically appraised for all peer reviewed manuscripts that suitably fulfilled the inclusion criteria established *a priori*. The protocol for this systematic review was developed according to the *Preferred Reporting Items for Systematic review and Meta-Analysis Protocols*, and followed methods outlined in *The Cochrane Handbook for Systematic Reviews of Interventions*. This systematic review is registered with PROSPERO. It is vital to consider diagnoses other than medullary thyroid carcinoma when presented with a patient with raised calcitonin, as it is not pathognomonic of medullary thyroid carcinoma. Lung neuroendocrine neoplasms can appear similar to medullary thyroid carcinoma histologically, they can secrete calcitonin and metastasize to the thyroid. Patients with medullary thyroid carcinoma may show stimulated calcitonin values over two or more times above the basal values, whereas calcitonin-secreting neuroendocrine neoplasms may or may not show response to stimulation tests. The present review summarises existing evidence from cases of ectopic hypercalcitonaemia to lung neuroendocrine neoplasms.

## Introduction

Neuroendocrine neoplasms (NENs) are a group of tumours that can often synthesise and secrete biologically active substances. This can lead to an array of clinical presentations, also referred to as paraneoplastic syndromes (PNS) (1).

PNS can occur as either the neoplasms acquire the ability to secrete a variety of biologically active substances, or by cross-reactivity between neoplastic and normal tissue (1, 2, 3, 4). The secretions from neoplasms can be eutopic, meaning from an expected site of origin which would usually secrete such a substance, or ectopic, referring to cells that would not normally be associated with secretion of that substance. The ectopic secretion of biological substances such as peptides, amines or cytokines isec more commonly seen by neoplasms that are formed in the gut (2, 4, 5). Endocrine PNS is the term used when PNS is from the ectopic secretion of a hormone, and can lead to diagnostic uncertainty, as it can manifest with symptoms identical to a neoplasm in the expected site of origin (4, 6, 7).

Calcitonin (CTN) is secreted from parafollicular cells located in the thyroid gland, but also can be present in the lung, bladder, small intestine, liver, thymus and parathyroid glands. Parafollicular cells are part of the neuroendocrine system and originate from primordial C cells (8, 9). The gene *CALC-1* creates procalcitonin, which is cleaved by a convertase enzyme to create CTN (5). In *in vitro* experimental models, CTN has a temporary effect on impairing osteoclast function by reducing its motility, inhibiting carbonic anhydrase II and preventing the development of mature osteoclasts. This, along with its effects on tubular epithelium within the kidney, reduces serum calcium and phosphate levels (8). However, the significance and biological role of CTN remains elusive.

High serum CTN concentrations may be suggestive of MTC but are not pathognomonic (9). MTC is a NEN originating from the thyroid and associated with eutopic secretion of CTN. However, CTN can also be raised due to chronic renal failure, pernicious anaemia, hepatic cirrhosis, various medications, extra-thyroid neoplasms, false-positive CTN assay laboratory results, lower respiratory tract infections, smoking, chronic inflammatory conditions of the lung, and any condition increasing gastrin or calcium such as hyperparathyroidism or Zollinger-Ellison syndrome (7, 8, 10, 11). These conditions tend to cause a moderate rise in CTN compared to MTC. Extra-thyroidal NENs, however, lead to a diagnostic dilemma as they can be associated with similar concentrations of CTN to MTC. Extra-thyroidal sources associated with abnormal CTN concentration have been observed in NENs within the pancreas, parathyroid glands, larynx, oesophagus, thymus, lung, small intestine, liver, bladder and adrenals, all of which can resemble MTC histologically (3, 5, 10, 12).

Around 1% of normal pulmonary tissue is made up of neuroendocrine cells, which can be solitary cells throughout the lung or form in clusters called neuroepithelial bodies (13, 14, 15). They remain in adult lungs, and continue to secrete bioactive substances such as calcitonin, calcitonin gene-related peptide, serotonin, chromogranin A, and gastrin-related peptide (15, 16, 17). Physiological stimuli, such as hypoxia, can lead to such agents being secreted (16), which may be why Machens *et al.* report fluctuations of hypercalcitonaemia in patients with chronic lung disease (10). There is speculation that neuroendocrine cells are the origin of lung NENs, but as we do not yet have evidence of the events of early cell change into neoplasm, this cannot be claimed definitively (16). However, around 25% of lung carcinomas (mainly small and large cell lung carcinomas) can develop neuroendocrine features and may be associated with endocrine PNS (7, 14). The development of an endocrine PNS may be what initially leads to the diagnosis of a carcinoma, or its recurrence in cases of patients with known malignancy (4, 18).

Extra-thyroidal CTN-secreting NENs can be indistinguishable from MTC in terms of immunohistochemical and biochemical profiles (4, 6). Due to the above similarities, biopsies with immunohistochemical characteristics of MTC, along with serum hypercalcitonaemia, could lead to an incorrect diagnosis of MTC. There have been cases of inappropriate thyroidectomy performed in patients with hypercalcitonaemia of extra-thyroidal origin due to concern of MTC (6, 8). We now report the diagnostic work-up of a patient found to have a lung lesion and a biopsied gluteal mass with histological appearances of MTC, in the context of morphological thyroid gland abnormalities and concurrent hypercalcitonaemia, and review the relevant literature.

### Methodology

This protocol was developed according to the *Preferred Reporting Items for Systematic review and Meta-Analysis Protocols* (PRISMA-P), and followed methods outlined in The Cochrane Handbook for Systematic Reviews of Interventions (15). This systematic review has been registered with PROSPERO (International Prospective Register of Systematic Reviews) with registration number CRD42021228917.

### Search strategy

Two reviewers (D L and G K D) conducted systematic searches of the following databases: Medical Literature Analysis and Retrieval System Online (MEDLINE), Excerpta Medica (EMBASE), Cochrane Central Register of Controlled Trials (CENTRAL), ClinicalTrials.gov, SCOPUS databases. Our key MeSH (Medical Subject Heading) search terms were; *“paraneoplastic” OR “ectopic” OR “neuroendocrine” AND “calciton…” AND “lung”*.

Moreover, reference lists of selected articles and other literature sources were browsed to ensure a comprehensive literature search was completed. None of the database searches filtered results based on year of publication date, and the last search was carried out in January 2021.

### Study selection

Expert opinion manuscripts, letters to the editor, commentaries, conference papers, animal studies, meta-analyses, and articles not in English, were excluded. Data were only included on adults (18 years or older), men and non-pregnant women. Articles were included if they examined from a lung lesion. No restrictions were made regarding the intervention type where a study took place, the number of participants or the duration of follow up. Covidence systematic review software (*Veritas Health Innovation, Melbourne, Australia*; http://www.covidence.org) was used for manuscript screening and extraction. Publications were initially screened for any duplicates before being assessed independently and in parallel by two reviewers. Any conflicts regarding the inclusion of a study were met with discussion and consensus. If an agreement had not been reached, arbitration by a third reviewer was utilised.

### Data extraction

Data were extracted independently by two reviewers following the *Cochrane Public Health Group Data Extraction and Assessment Template* to construct our own data extraction template that was pilot-tested and systematically used for each article. Data extracted included; study description (e.g. title, primary author, publication year, type of study, number of participants, type of lung CTN producing neoplasm and follow-up duration). The primary outcome was the incidence of calcitonin-secreting lung NENs.

### Quality assessment

Each study was assessed for bias using the Newcastle-Ottawa scale for observational studies. Studies were evaluated on eight factors, categorised into three groups: selection (including whether the cohort is representative of the population), comparability (assessed on grounds of study design and the analysis performed) and outcome (i.e. the assessment of outcome, follow-up rate and adequacy follow-up period).

Stars were awarded per category, with a maximum of four, two and three stars possible for the ‘selection’, ‘comparability’ and ‘outcome’ categories respectively. By following the guidelines of the Newcastle-Ottawa scale (16), two reviewers assessed the studies to be of poor (3 stars or less), fair (4-6 stars) or good (7-9 stars) quality.

### Data synthesis and statistical analysis

Heterogeneity was visually inspected, and due to the high variability and insufficient data results were not pooled into a meta-analysis, and a narrative synthesis was conducted instead.

## Results

Our advanced search produced 327 manuscripts that were imported for screening against predefined criteria. There were no duplicate studies and all 327 manuscripts were screened against title and abstract which resulted in 29 manuscripts progressing to a full-text assessment. A further 6 manuscripts were excluded as they were not in English, resulting in 23 eligible manuscripts.

**Figure 1:**
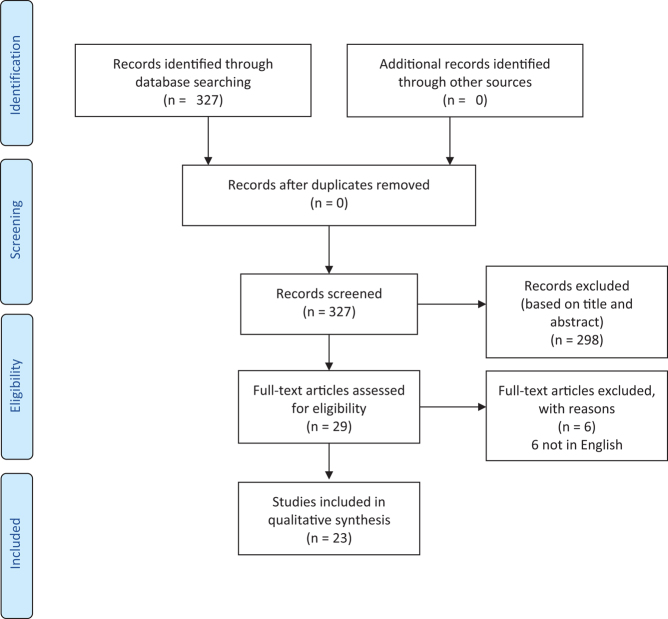
Flow diagram demonstrating the breakdown of the screening process.

*The Preferred Reporting Items for Systematic Reviews and Meta-Analyses* (PRISMA-P) flow diagram is shown in Fig. 1, outlining the outcomes of the screening proce

## Case report

A 72-year-old Caucasian female presented in January 2020 with weight loss and a gluteal mass. She had no major co-morbidities and was not on any regular medications. She had noted gradual weight loss which had started since August 2019, with recurrent ear infections. She suffered with intermittent loose stools but no flushing, later diagnosed as terminal ileitis.

**Figure 2:**
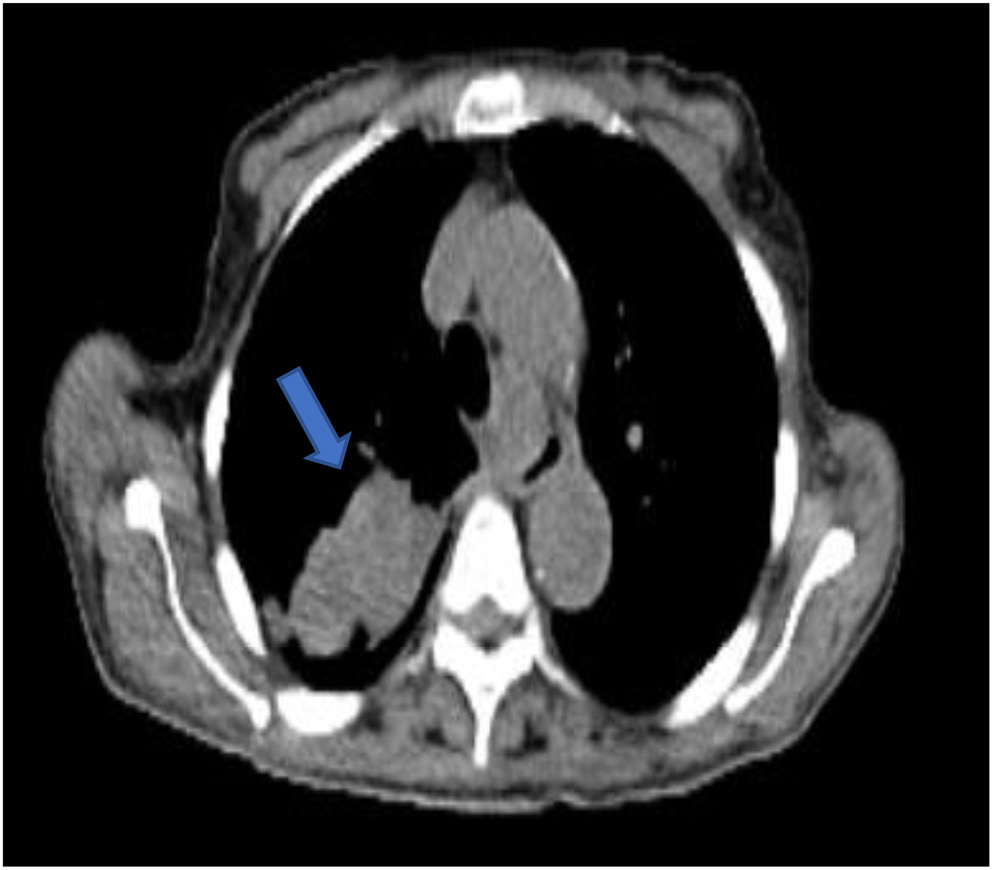
CT scan with transverse plane view of the 4 × 4.5 cm right upper lobe lung lesion.

She subsequently had a chest, abdomen and pelvis (CAP) contnrast-enhanced CT which showed bilateral pulmonary emboli, and a large 4 × 4.5 cm mass within the right upper lobe of the lung (Fig. 2). Furthermore, there was evidence of a large retrosternal thyroid goitre and no further evidence of any intra-abdominal abnormalities. An enhancing lesion in the left gluteus was seen, thought to be in keeping with a soft tissue metastasis.

**Figure 3:**
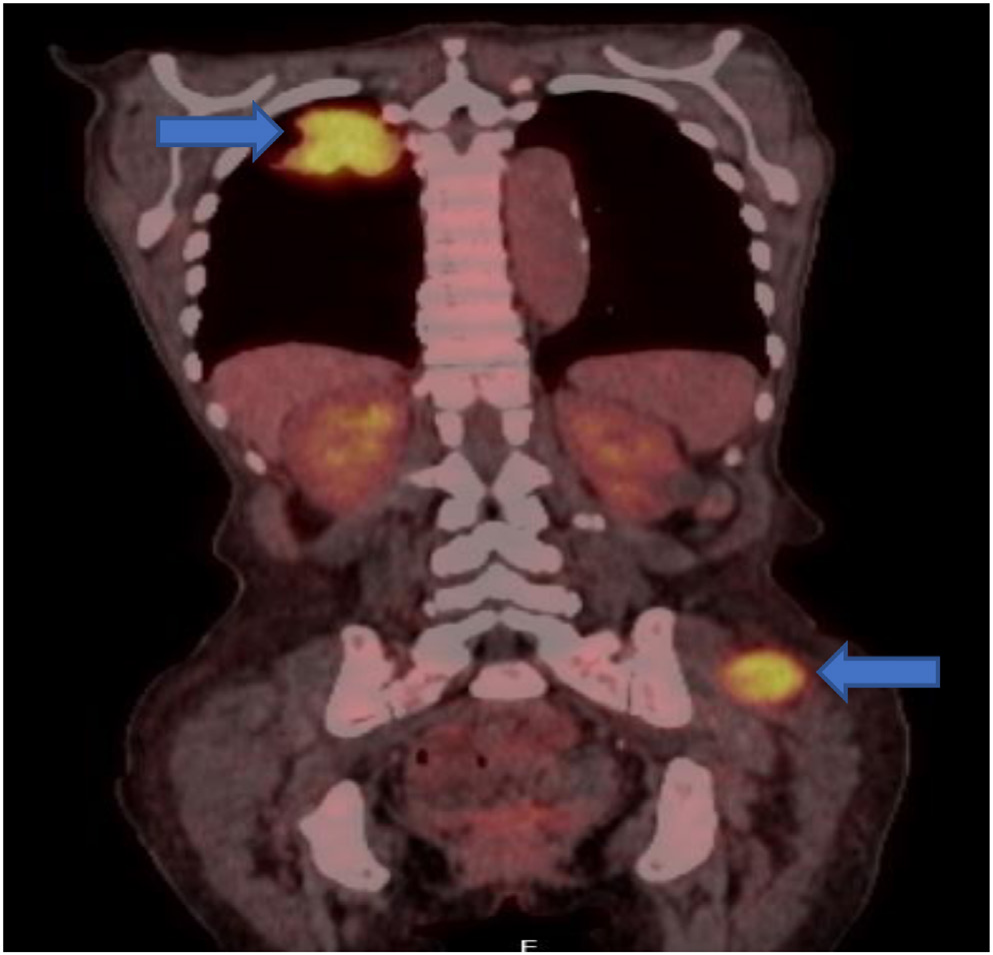
^18^FDG-PET coronal CT displaying two avid lesions, in the right upper lobe and left gluteal region.

She was referred for a ^18^fluoro-deoxyglucose PET (^18^FDG-PET) which showed normal metabolic activity throughout the thyroid, including the retrosternal goitre. There was intense uptake (SUV max >4.5) within the 4.5cm right upper lobe lung and gluteal mass (Fig. 3).

**Figure 4:**
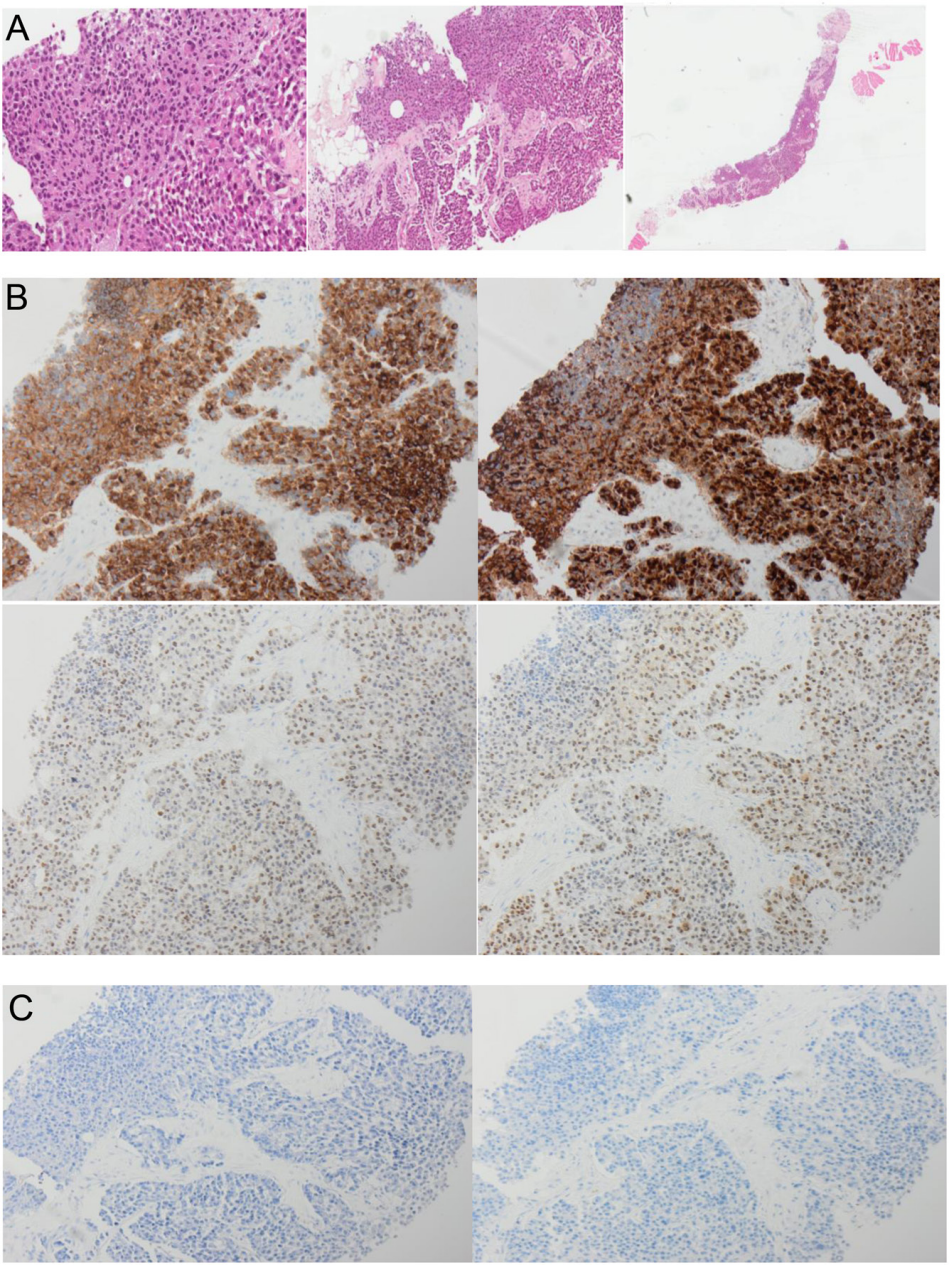
(A) Haematoxylin & eosin staining of the left gluteal lesion composed of sheets of cells with abundant eosinophilic cytoplasm and eccentric nuclei with stippled chromatin, some with nuclear pseudo-inclusions. Twenty-five mitotic figures/2 mm slides. (B) Positive immunoperoxidase staining, ×100 magnification, A: Synaptrophysin, B: Calcitonin, C: PAX8, D: TTF1. (C) Negative immunoperoxidase staining, ×100 magnification, A: thyroglobulin, B: chromogranin.

A biopsy of the left gluteal lesion showed fibro-fatty tissue infiltrated with malignant cells arranged predominantly with solid sheets. The cells had abundant eosinophilic cytoplasm and eccentric nuclei with frequent nuclear inclusions noted (Fig. 4A). Malignant cells were positive for cytokeratin AE1/3, TTF1, PAX-8, synaptophysin and calcitonin, with granular staining for CEA (Fig. 4B). Staining for chromogranin A, S100, Melan A, CD45, Napsin A, thyroglobulin and p40 was negative (Fig. 4C). The Ki-67 proliferation index was high at 84%. The morphology and immunophenotype were most in keeping with metastatic medullary thyroid carcinoma, although other metastatic neuroendocrine neoplasms could not be completely excluded.

**Figure 5:**
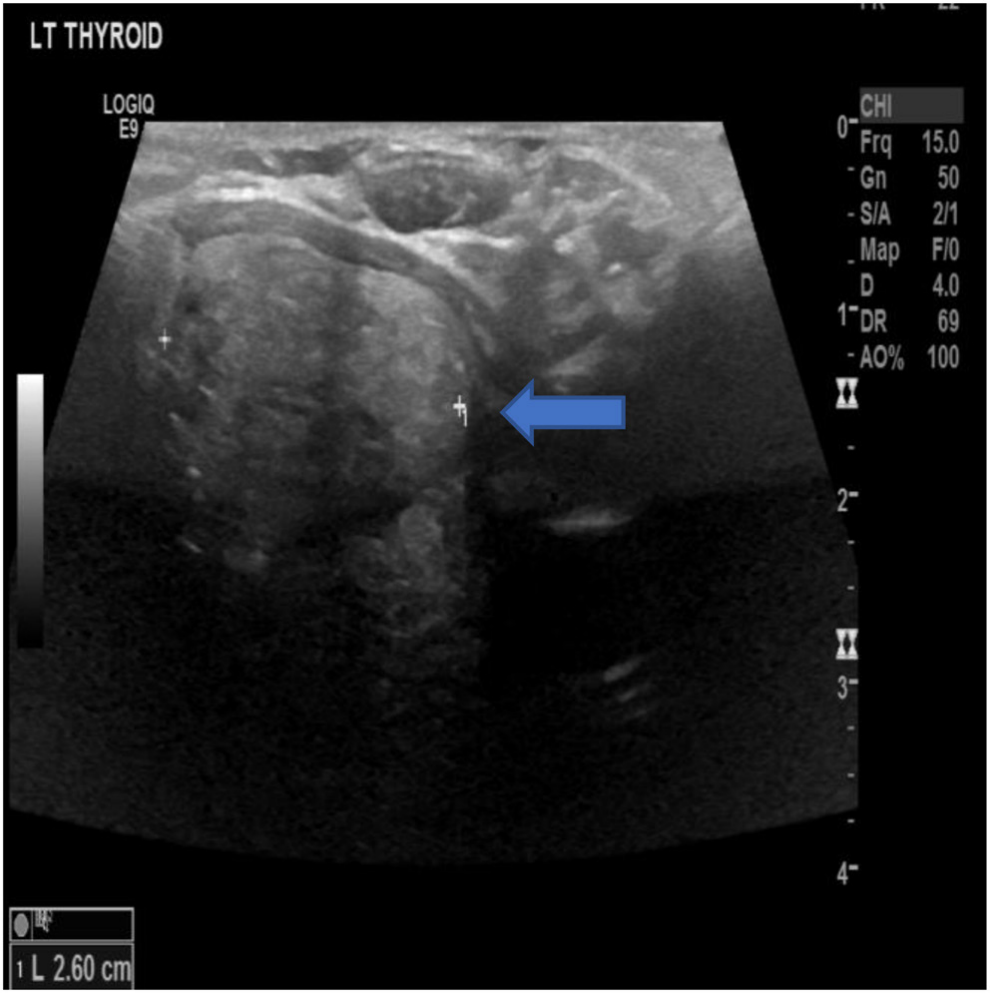
Thyroid USS demonstrating a 2.6 cm left confluence of spongiform thyroid nodules, with hypoechoic halos (U2). No cervical lymphadenopathy.

She was also referred for a neck ultrasound scan (USS) which showed a 2.6cm left confluence of spongiform thyroid nodules, with hypoechoic halos (U2). There was no cervical lymphadenopathy (Fig. 5).

Following discussion in the thyroid cancer multi-disciplinary team meeting, her left thyroid confluence was upgraded to U4 using British Thyroid Association (BTA) guidelines (Table 1) (12). Serum calcitonin was measured and fine-needle aspiration (FNA) of the thyroid confluence was performed to help rule out MTC. Using BTA classification, cytology was in keeping with a Thy-2 sample, which maps directly to the American Bethesda Categories and means it was non-neoplastic (Table 2) (12).

**Table 1:** U grading of thyroid nodules based on ultrasound imaging (18).

U1	U2	U3	U4	U5
Normal	Benign	Indeterminate/equivocal	Suspicious	Malignant
Normal thyroid tissue	Halo	Homogenous	Solid	Hypo-echoic
	Iso-echoic or mildly hyperechoic	Hyper-echoic solid, halo (follicular lesion)	Hypo-echoic or very hypo-echoic	Lobulated or irregular outline
	Cystic change ± ring		Disrupted peripheral calcification lobulated outline	Micro-calcific
	Down sign micro-cystic/spongiform. Peripheral	Equivocal echogenic foci		
	Egg shell calcification	Cystic change mixed/central vascularity		Globular calcification
	Peripheral vascularity			Intra-nodular vascularity Shape (taller > wide) Characteristic-associated lymphadenopathy
No follow-up required	No follow-up required – routine FNAC not recommended unless high clinical suspicion of thyroid cancer	FNAC	FNAC	FNAC

FNAC, fine-needle aspiration cytology.

**Table 2:** Thy diagnostic grading for fine-needle aspiration cytology (FNAC) (18).

Thy1	Thy2	Thy3		Thy4	Thy5
		Thy 3F	Thy 3A		
Non-diagnostic	Non-Neoplastic for example, colloid nodule or thyroiditis	Follicular lesion	Atypia present	Suspicious of thyroid cancer	Diagnostic of thyroid cancer
Repeat FNAC	No follow-up if no suspicious US features and no clinical suspicion of thyroid cancer	Diagnostic hemithyroidectomy* Consider total Thyroidectomy in lesions >4 cm where the incidence of malignancy is higher	Repeat ultrasound and FNAC If second Thy 3 A cytology obtained, discuss at MDT and consider diagnostic hemithyroidectomy	Discuss at MDT Diagnostic hemithyroidectomy	Discuss at MDT Appropriate further investigations for staging where indicated Total thyroid-ectomy ± node clearance in appropriate high-risk patients

FNAC, fine-needle aspiration cytology.

Calcitonin was 3900 pg/mL (normal range 0–4.8), CEA 4.6 µg/L (normal range 0–3.8). She was reviewed in the clinic, by which time she had already been on prednisolone 10 mg daily to try and stimulate her appetite. While in clinic, she reported significant polydipsia and polyuria, and her serum blood glucose was 21 mmol/L. She was admitted for urgent inpatient management of steroid-induced diabetes.

**Figure 6:**
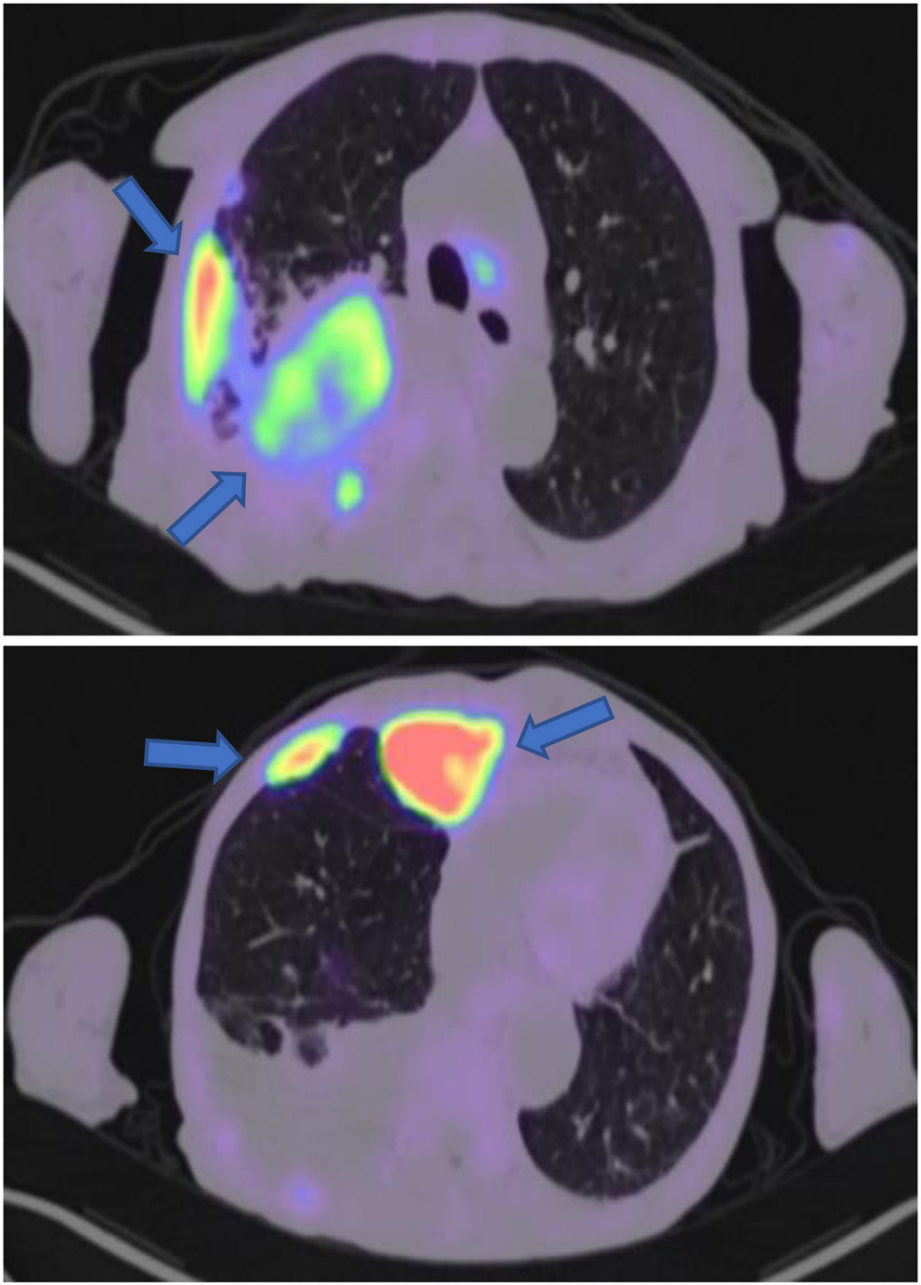
^68^Ga-DOTATATE PET/CT scan with arrows pointing to the upper lobe primary, pleural and nodal disease.

While an inpatient, she was also referred for a ^68^Ga-DOTATATE PET/CT scan which showed multiple sites of progression with pleural and nodal disease (Fig. 6). The finding was concerning of an upper right lobe lung primary with extensive right pleural and possibly solitary left pleural, nodal (above and below the diaphragm), and soft tissue (posterior to left psoas muscle ad inter left gluteal muscle) metastases.

It was decided that, considering the results of gluteal mass histology, thyroid nodule cytology and imaging, the most likely diagnosis was not MTC but that of a neuroendocrine lung neoplasm, grade 3. This patient deteriorated rapidly and died of complications before any further interventions were possible.

## Discussion

CTN-secreting NENs are rare, with few reported cases in the last 20 years. While small cell lung carcinomas (SCLC) are more commonly associated with ectopic CTN secretion, a review by Bondy *et al.* suggested that in 51% of cases it is secondary to SCLC, 20% of squamous cell, 33% to adenocarcinoma and 33% to large cell lung carcinomas (LCLC) (19). As patients with ectopic CTN secretion may not display overt clinical symptoms, they might not be tested for serum CTN and this may be one reason for the low number of reported cases. Another reason for the low number of reported cases may be that raised CTN is not associated with characteristic symptoms distinguishing this from other PNS.

Reported symptoms associated with raised CTN could be diarrhoea, flushing, metabolic alkalosis with hypokalaemia, hypocalcaemia, hypomagnesaemia, hypophosphataemia and hyperglycaemia (3, 20). Nozières *et al.* showed that only one-third of the patients with significantly raised CTN concentration above 100 ng/L had symptoms such as profuse diarrhoea and deranged electrolytes such as hypokalaemia, hypophosphataemia, hypocalcaemia and metabolic alkalosis (4). There are multiple other neuroendocrine neoplasms causing diarrhoea such as gastrinomas, VIPomas, glucagonomas, somatostatinomas and the carcinoid syndrome, as well as medullary thyroid cancer and hypercalcitonaemia secreted from the pancreas causing Verner-Morrison syndrome (20). In the reported cases, when patients with CTN-secreting lung NENs have clinical symptoms, there is usually co-secretion of other biologically active substances, and it is these substances that most likely explain the symptoms rather than the raised CTN (2, 3, 5, 7, 20).

Evidence that the frequency of hypercalcitonaemia may be higher than expected comes from Tsutsumi *et al.*, who found 10 out of 45 SCLC patients were CTN immunoreactive, and 14 out of 32 patients with bronchial carcinoid had CTN immunoreactivity on histopathological examination (21). Interestingly, their immunohistochemical studies demonstrated that bronchial carcinoids appear to imitate foetal or neonatal lung in terms of calcitonin/calcitonin gene-related peptide expression. However, it was not known whether these patients had raised serum CTN concentrations. A significant proportion of lung neoplasms from that study were tested histologically and appeared to have the ability to express CTN, albeit patients were more often clinically asymptomatic (21).

A summary of the study characteristics that were included in our systematic review are included in Table 3. It is important to note that the most significant studies were performed before 1995: manuscripts published after 1995 are mostly case reports or small case series. A possible explanation for this is the method CTN was tested in the past. The initial approach was using RIA, which lacked sensitivity and specificity, and so this led to a two-sided IRMA developed in the late 1980s (8). In the early 2000s, there was a movement to fluorescent and chemiluminescent tests, which were more accurate. Enzyme-linked immunoassay (ELISA) technique was used, but an issue with ELISA was heterophilic antibody interference, associated with false positives or higher than actual values (8). One of the latest developments is the electrochemiluminescence immunoassay (ECLIA), which allows for a prompt and accurate diagnosis (8). This may be a reason why when large cohorts of patients with various NENs are randomly screened for CTN concentrations, such as with Daskalakis *et al.* (2), the percentage of them with hypercalcitonaemia is considerably less than several decades ago.

**Table 3:** Characteristics of CTN-secreting lung NEN studies.

Study	Country	Type of neoplasm	Study design	Follow-up (Months)	Sample size (n)	Calcitonin value (ng/L)	Symptomatic calcitonaemia	Assessment of outcome	NOS Star Award
Coombes *et al*. (22)	UK	All SCLC	Prospective	Not mentioned	t	>100	Not mentioned	Increased CTN concentrations of not diagnostic of MCT	8
Ellison *et al*. (23)	UK	Likely poorly differentiated squamous carcinoma	Case Report	18 months	1	2580	No	Cells *in vitro* had a stable heritable characteristic of immunoreactive CT synthesis and release.	NA
Hillyard *et al*. (24)	UK	All SCLC	Prospective	Not followed-up	31	2500, 2200, 890, 4900	Not mentioned	That lung could cause ectopic production of CTN	8
Hansen *et al*. (37)	Denmark	38% adenocarcinoma, 29% SCLC, 22% squamous cell, 13% LCLC	Prospective	Not followed-up	79	109	Not mentioned	CTN frequently elevated in SCLC, and a positive pentagastrin test is not pathognomonic for MTC	8
Silva *et al*. (25)	USA	All SCLC	Prospective	Not mentioned	61	Concentrations had to be >300, range 300 to 17500	Not mentioned	CTN may be a useful marker to assess results of therapy in bronchogenic cancer. 52% had raised CTN	7
Hansen *et al*. (27)	Denmark	All SCLC	Prospective	Not mentioned	74	Up to 55000	Not mentioned	Bone metastases have no influence on CTN concentrations. Results resemble concentrations seen in MTC. 2/3 patients had raised CTN	8
Hansen *et al*. (26)	Denmark	All SCLC	Prospective	Not followed-up	75	1300	Not mentioned	CTN is not correlated with stage of disease	8
Hansen *et al*. (28)	Denmark	All SCLC	Prospective	Not mentioned	75	Mean value 1300	Not mentioned	CTN raised in 65% of their patients. No significant difference to stage of disease, response to treatment or survival	7
Study	Country	Type of neoplasm	Study design	Follow-up (Months)	Sample size (n)	Calcitonin value (ng/L)	Symptomatic calcitonaemia	Assessment of outcome	NOS Star Award
Hansen *et al*. (29)	Denmark	All SCLC	Prospective	18 months	3 parts to experiment, n=6,12,29	Concentrations > 79	Not mentioned	Initial response to treatment, decrease with treatment when there is disease regression. Moderate increase to CTN with relapse, albeit disease symptoms clinically significant and so deemed not useful for relapse	9
Gropp *et al*. (30)	Germany	All SCLC	Prospective	Not followed-up	110	Concentrations had to be above 125	Not mentioned	CTN elevated in 26/54 SCLC, 4/41 squamous cell, 2/19 large cell. CTN concentrations dropped with treatment and rise if a poor response. Does not correlate with stage of disease	8
Roberts *et al*. (33)	USA	38% adenocarcinoma, 29% SCLC, 22% squamous cell, 13% LCLC	Case report	Died before treatment commenced	1	>1000	Not mentioned	Rapidly deteriorated and died before treatment	N/A
Tabolli *et al*. (32)	Italy	All SCLC	Prospective	Not followed-up	41	>57 females, >97 males	Not mentioned	As concentrations were often higher in people with cancer, it was felt to be a good marker	8
Sano *et al*. (34)	Japan	All SCLC	Case report	6 months	1	340	No	CTN returned to normal after surgical resection. However, suggestive of bone metastasis 6 months after surgery	N/A
Samuels *et al*. (36)	Canada	All SCLC	Retrospective	Not mentioned	127	Mean value 1300	Up to 999, but majority <500	No significant difference with CTN concentrations and stage of malignancy or survival	8
Kelley *et al*. (10)	USA	All SCLC	Prospective	Not mentioned	86	concentrations > 79	647	CTN nit a good marker for treatment response. CTN raised if a smoker	7
Monsieur *et al*. (17)	Belgium	Adenocarcinoma	Case report	Died before treatment commenced	1	Concentrations had to be > 125	65500	Rapid deterioration and died, treatment not commenced	N/A
Eagle *et al*. (3)	USA	Carcinoid	Case report	6 months	1	>1000	165	Metastasis from lung neoplasm to the thyroid	N/A
Machens *et al*. (9)	Germany	All SCLC	Prospective	Not followed-up	13	>57 females, >97 males	42.9, 43.4, 44.7 and 585.0	Pentagastrin stimulation test can lead to significant rises in MTC in non-MTC	7
Pratz wt al (20)	USA	LCLC	Case report	Died 2 weeks after treatment commenced	1	340	9571	Cisplatin and etoposide saw 2-week transient improvement but patient then deteriorated and died	N/A
Coners *et al*. (3)	USA	SCLC	Case report	Died after 1st cycle of treatment	1	81.8	Diarrhoea thought not to be due to CTN	Somatostatin, cisplatin and etoposide saw a moderate improvement to CTN concentrations, but patient deteriorated and died after 1st cycle of treatment	N/A
C vijovic *et al*. (6)	Serbia	LCLC	Case report	Patient died after 7th cycle of treatment	1	260	No	6 cycles of cisplatin and etoposide saw a moderate improvement to CTN concentrations. One cycle of Peptide Receptor Radionuclide Therapy, but patient deteriorated and died	N/A
Study	Country	Type of neoplasm	Study design	Follow-up (Months)	Sample size (n)	Calcitonin value (ng/L)	Symptomatic calcitonaemia	Assessment of outcome	NOS Star Award
Nozières *et al.* (4)	France	Study does not specify type, 4 out of 17 were documented as carcinoid	Retrospective	5.6 years	176	>100	One third of the patients with CTN >100pg/ml were potentially symptomatic	8 out of 11 patients who were followed up had highest CTN concentration before death. 4 of these 11 patients had lung neoplasms, all of which carcinoid. The trend towards poor survival if high CTN was not significant. No correlation with CTN and staging.	7
Vahidi *et al.* (5)	USA	Atypical carcinoid	Case report	Not discussed	1	5900	No	Important to consider differentials, in case of unnecessary thyroidectomies	N/A
Daskalaskis *et al.* (1)	Sweden	Carcinoid	Retrospective	Mean follow up 89.6 months	834	Unknown but had to be >10 to be included in study	Potentially, but could be explained by short colonic transit times	All 4 patients with raised CTN had disseminated disease at diagnosis. All 4 were described together with another endocrine PNS	8

SCLC, small cell lung carcinoma; CTN, calcitonin; LCLC, large cell lung carcinoma; MTC, medullary thyroid carcinoma.

Coombes *et al.*, Ellison *et al.* and Hillyard *et al.* published some of the earlier studies demonstrating that lung carcinomas can lead to ectopic secretion of CTN (22, 23, 24). Coombes *et al*. showed that, in 8 out of 11 patients with SCLC in their study, there was ectopic CTN secretion (22).

Silva *et al.* showed that 52% of their 61 patients had hypercalcitonaemia (25). They also demonstrated that if the patient had a clinical response to treatment, this would be mirrored by a decrease in CTN concentration. Hansen *et al.* measured CTN concentrations in 75 patients with SCLC and found elevated CTN in 48 patients (26). Multiple patients in this study also had co-secretion of either ACTH or vasopressin (ADH). Hansen *et al*. found that there was no correlation with CTN and stage of disease, meaning higher CTN concentrations were not associated with more extensive disease (26).

Hansen *et al.* questioned whether previous studies reporting on the incidence of hypercalcitonaemia in their cohorts was due to infiltrative bone disease (27). None of the 74 patients from their cohort had hypercalcaemia, albeit two-thirds had hypercalcitonaemia. They found that when comparing different stages of the disease, there was no significant difference in CTN concentration, and that bone metastases did not lead to CTN elevations. CTN concentrations were in the range expected for MTC. In another study, Hansen *et al.* found that CTN concentration was not predicting response to treatment comparing responders to non-responders (28).

Hansen *et al.* performed a detailed follow-up of patients with hypercalcitonaemia (29). Their study had three arms and all patients in their study had SCLC. In the first arm, 6 patients with raised CTN concentrations had blood tests taken prior to their chemotherapy which consisted of cyclophosphamide and vincristine. Blood samples were then repeated at regular intervals over a week. 4 out of 6 patients had raised baseline CTN concentrations, and in those 4 patients there was a significant decrease in CTN levels at 1 week following treatment. The second arm had baseline blood tests prior to chemotherapy, 3–6 months later, and then again if there was evidence of disease relapse: 11 patients in this arm had baseline CTN concentrations exceeding 79 pg/mL and tumour partial or complete response to treatment. In this group, 9 of 11 patients who responded to treatment also had a significant reduction in CTN. Eight of these 9 patients then had tumour relapse, with a subsequent rise in CTN concentration. However, this rise was only moderate, albeit disease symptoms were clinically significant, indicating that CTN was not a useful prognostic marker. The results suggested that only in a small proportion of patients would CTN be of prognostic value, but no consistent patient characteristics were identified. Only three patients with hypercalcitonaemia in remission were followed up to 18 months after treatment initiation. Their CTN concentrations had only been moderately increased at baseline and remained stable after 18 months follow-up.

Gropp *et al.* used a higher pathological CTN cut off (>125 pg/mL), and abnormal results were observed in 26 out of 54 patients with SCLC. There was no positive or negative correlation to disease stage (30). CTN concentrations would decrease if there was a good response to treatment and rise in poor responders but findings were only numerically significant. In a study by Coombes *et al.*, while higher baseline serum CTN values were associated with a poorer response to treatment, it was recommended that measuring serum CTN to predict response to treatment should be kept to a minimum (31).

Tabolli *et al.* reported a high incidence of hypercalcitonaemia, with 53.6% of their 41 bronchogenic carcinomas showing a raised CTN, but unlike Gropp *et al*. they did not provide information regarding their histopathological characteristics (32). As concentrations were regularly increased in patients with malignant neoplasms, CTN was felt to be a useful marker.

Roberts *et al.* presented a case of a patient with hypercalcitonaemia and amylase co-secretion. The patient also had raised adrenocorticotrophic hormone (ACTH), raised growth hormone, and raised luteinising hormone (33). Sano *et al.* discuss a patient with lung carcinoid co-secreting somatostatin and CTN (34). A particularly rare case of ectopic co-secretion of six hormones including CTN from one tumour was reported by Monsieur *et al.* (17). Melanin production in MTC and lung carcinoid tumours leading to diagnostic uncertainty has been reported by Eagle *et al.* (35). Initially, the diagnosis was thought to be primary amelanotic choroidal melanoma with choroidal metastases. However, detailed investigations confirmed a primary lung carcinoid (35).

In a study of patients with untreated SCLC, Samuels *et al*. demonstrated no relationship between CTN concentration and disease stage or survival rate (36). On review of previous studies, they estimated that 25–66% of patients with SCLC have elevated CTN concentrations at the time of diagnosis; 71% of their 69 patients had elevated CTN, but the only significant result found was hypercalcitonaemia in liver metastases, with hypercalcitonaemia having a sensitivity of 100% but a specificity of only 41% and a positive predictive value of 40%. As with Hansen *et al.*, there was no significant difference in CTN concentrations in case of metastatic bone disease (27).

Kelley *et al.* designed a study with 3 arms, including non-smokers without SCLC, smokers without SCLC, and smokers with SCLC (10). All but one of the 49 patients smoked in the SCLC group. They were able to demonstrate that in the two groups without SCLC, smoking appeared to moderately increase CTN. Kelley *et al.* also demonstrated in a murine animal model that air pollution or smoking increased the number of pulmonary neuroendocrine cells that were positive for CTN on immunohistochemical staining, and that smoking appeared to cause hyperplasia of the pulmonary neuroendocrine cells. In their human study, CTN concentrations were not correlated with the severity of smoking. Interestingly, 6 patients with SLCL had negative staining for CTN on immunohistochemistry, but elevated serum CTN. Like in other cases of PNS with peripherally raised hormone but negative staining, one theory for this may be that SCLC cells may have acquired the ability to secrete CTN but have not completely altered their machinery to express this histologically. It was concluded that the use of CTN was not a reliable marker for monitoring treatment response (10).

Interestingly, on immunohistochemical studies of 2 of the reported studies reported in Table 3, there was negative CTN tissue staining but raised serum levels, similar to the findings of Kelley *et al.* (10, 11, 20). Pratz *et al*. (20) hypothesised that large cell lung carcinoma (LCLC) cases they described co-secreted VIP and CTN. One theory was that LCLC tumours from their study were biologically behaving like a VIPoma, in which cells rapidly secrete CTN reaching high serum concentrations, and so may not be seen expressed within the tumour cells. An alternative theory was that the neoplasm secretes an as yet unknown substance that stimulates CTN secretion from C cells. Whilst there can be other causes of hypercalcitonaemia (7, 8, 10), these would not induce CTN elevation as such reported by Pratz *et al.* (20).

Coners *et al.* reported the first case of SCLC co-secreting ACTH and CTN (3). Cvijovic *et al.* presented a patient with multinodular goitre, neck lymphadenopathy, raised CTN and raised carcinoembryonic antigen (CEA), who was referred to them initially with the diagnosis of MTC (7). Surgical excision of the lymph node showed this to be a metastasis from a neuroendocrine neoplasm, with bronchoscopy confirming the diagnosis (7).

Similarly, Vahidi *et al.* report a case of suspected MTC but with an incidental lung lesion seen on a chest x-ray and biopsy suggesting either MTC or other NEN. Both cases by Cvijovic *et al*. and Vahidi *et al.* are similar to our case as thyroid FNA was required to help rule out MTC (6, 7).

Daskalaskis *et al.* performed a large retrospective study of more than 700 patients with NENs to describe the incidence of endocrine paraneoplastic syndromes (2). Only four patients were found to have hypercalcitonaemia, but the study included patients with multiple NEN sites not just originating from the lung. One of these patients was documented as having a lung carcinoid. All four patients had stage IV disease, and all four were described as having concurrently other endocrine PNS with hypercalcitonaemia. This study highlights the rarity of lung NENs-secreting CTN (2).

Machens *et al.* and Hansen *et al.*, focused on the use of CTN stimulation tests to look at whether CTN-secreting NENs can be distinguished from MTC based on response to calcium or pentagastrin tests. Whilst Machens *et al.* suggested that MTC tends to be associated with a doubling CTN concentration following stimulation, Hansen *et al.* warned that there can still be a significant CTN rise in lung NENs, and that high CTN response to stimulation is not pathognomonic for MTC (9, 37). In this study, 54 of the 79 patients had hypercalcitonaemia prior to pentagastrin test, with 25% of them having CTN concentrations expected of MTC.

In the largest recent study of CTN-secreting NENs, Nozières *et al.* reviewed the data of 17 patients with lung NENs-secreting CTN (5). Nine of these patients had high CTN concentrations over 100 ng/L at diagnosis. Whilst Nozières *et al.* did not demonstrate an association between CTN concentrations and prognosis, patients whose CTN concentration was >100 ng/L had higher grade neoplasms. They suggest testing for CTN in patients with foregut NENs and diarrhoea (5).

## Conclusions

This work highlights the importance of considering differentials to MTC when presented with thyroid pathology and hypercalcitonaemia, and that although raised serum calcitonin suggests MTC, it is not pathognomonic. Our review of the literature highlights conflicting results when interpreting CTN role in predicting biological behaviour, response to treatment or overall survival rates in cases of patients with ectopic hypercalcitonaemia to lung NENs. The interpretation of elevated serum calcitonin is still a matter of debate given the many confounding factors, variety of assays with limited efficacy and different cut-offs. We propose a thorough clinical evaluation of all patients presenting with elevated CTN concentration followed by multidisciplinary discussion when planning investigations and therapy to avoid misdiagnosis of ectopic CTN sources of secretion.

## Declaration of interest

The authors declare that there is no conflict of interest that could be perceived as prejudicing the impartiality of the research reported.

## Funding

National Institute for Health Research (NIHR), South London Clinical Research Network (CRN) Strategic ‘Green shoots’ Investigator Award supported Dr Dimitriadis in the writing of this manuscript.

## References

[1] Dimitriadis GK Angelousi A Weickert MO Randeva HS Kaltsas G Grossman A Paraneoplastic endocrine syndromes. Endocrine-Related Cancer 2017 24 R173–R190. (10.1530/ERC-17-0036) 28341725

[2] Daskalakis K Chatzelis E Tsoli M Papadopoulou-Marketou N Dimitriadis GK Tsolakis AV Kaltsas G Endocrine paraneoplastic syndromes in patients with neuroendocrine neoplasms. Endocrine 2019 64 384–392. (10.1007/s12020-018-1773-3) 30280284 PMC6531606

[3] Coners K Woods SE Webb M Dual paraneoplastic syndromes in a patient with small cell lung cancer: a case report. Journal of Medical Case Reports 2011 5 318. (10.1186/1752-1947-5-318) PMC3158120 21771301

[4] Nozières C Chardon L Goichot B Borson-Chazot F Hervieu V Chikh K Lombard-Bohas C Walter T Neuroendocrine tumors producing calcitonin: characteristics, prognosis and potential interest of calcitonin monitoring during follow-up. European Journal of Endocrinology 2016 174 335–341. (10.1530/EJE-15-0917) 26671974

[5] Vahidi S Stewart J Amin K Racila E Li F Metastatic medullary thyroid carcinoma or calcitonin-secreting carcinoid tumor of lung? A diagnostic dilemma in a patient with lung mass and thyroid nodule. Diagnostic Cytopathology 2018 46 345–348. (10.1002/dc.23852) 29124912

[6] Cvijovic G Micic D Kendereski A Zoric S Sumarac-Dumanovic M Tatic S Trivic A Pejkovic-Stamenkovic D Jeremic D Ectopic calcitonin secretion in a woman with large cell neuroendocrine lung carcinoma. Hormones 2013 12 584–590. (10.14310/horm.2002.1447) 24457407

[7] Giannetta E Guarnotta V Altieri B Sciammarella C Guadagno E Malandrino P Puliani G Feola T Isidori AM Colao AALet al. ENDOCRINE TUMOURS: Calcitonin in thyroid and extra-thyroid neuroendocrine neoplasms: the two-faced Janus. European Journal of Endocrinology 2020 183 R197–R215. (10.1530/EJE-20-0506) 33112280

[8] Gambardella C Offi C Clarizia G Romano RM Cozzolino I Montella M Di Crescenzo RM Mascolo M Cangiano A Di Martino S Medullary thyroid carcinoma with double negative calcitonin and CEA: a case report and update of literature review. BMC Endocrine Disorders 2019 19 103. (10.1186/s12902-019-0435-7) PMC6794852 31619220

[9] Machens A Haedecke J Holzhausen HJ Thomusch O Schneyer U Dralle H Differential diagnosis of calcitonin-secreting neuroendocrine carcinoma of the foregut by pentagastrin stimulation. Langenbeck’s Archives of Surgery 2000 385 398–401. (10.1007/s004230000169) 11127524

[10] Kelley MJ Becker KL Rushin JM Venzon D Phelps R Ihde DC Bliss DP Melby K Snider RH Johnson BE Calcitonin elevation in small cell lung cancer without ectopic production. American Journal of Respiratory and Critical Care Medicine 1994 149 183–190. (10.1164/ajrccm.149.1.8111580) 8111580

[11] Ghillani PP Motté P Troalen F Jullienne A Gardet P Le Chevalier T Rougier P Schlumberger M Bohuon C Bellet D Identification and measurement of calcitonin precursors in serum of patients with malignant diseases. Cancer Research 1989 49 6845–6851. 2555054

[12] Sui P Wiesner DL Xu J Zhang Y Lee J Van Dyken S Lashua A Yu C Klein BS Locksley RM Pulmonary neuroendocrine cells amplify allergic asthma responses. Science 2018 360 eaan8546. (10.1126/science.aan8546) PMC6387886 29599193

[13] Naizhen X Linnoila RI Kimura S Co-expression of achaete-scute homologue-1 and calcitonin gene-related peptide during NNK-induced pulmonary neuroendocrine hyperplasia and carcinogenesis in hamsters. Journal of Cancer 2016 7 2124–2131. (10.7150/jca.16399) 27877229 PMC5118677

[14] Weichselbaum M Sparrow MP Hamilton EJ Thompson PJ Knight DA A confocal microscopic study of solitary pulmonary neuroendocrine cells in human airway epithelium. Respiratory Research 2005 6 115. (10.1186/1465-9921-6-115) PMC1277851 16216130

[15] Song H Yao E Lin C Gacayan R Chen MH Chuang PT Functional characterization of pulmonary neuroendocrine cells in lung development, injury, and tumorigenesis. PNAS 2012 109 17531–17536. (10.1073/pnas.1207238109) 23047698 PMC3491514

[16] Gosney JR Sissons MC Allibone RO Neuroendocrine cell populations in normal human lungs: a quantitative study. Thorax 1988 43 878–882. (10.1136/thx.43.11.878) 3065973 PMC461542

[17] Monsieur I Meysman M Noppen M de Greve J Delhove O Velckeniers B Jacobvitz D Vincken W Non-small-cell lung cancer with multiple paraneoplastic syndromes. European Respiratory Journal 1995 8 1231–1234. (10.1183/09031936.95.08071231) 7589410

[18] Mitchell AL Gandhi A Scott-Coombes D Perros P Management of thyroid cancer: United Kingdom National Multidisciplinary Guidelines. Journal of Laryngology and Otology 2016 130 (Supplement 2) S150–S160. (10.1017/S0022215116000578) PMC4873931 27841128

[19] Bondy PK The pattern of ectopic hormone production in lung cancer. Yale Journal of Biology and Medicine 1981 54 181–185. PMC2595956 6270916

[20] Pratz KW Ma C Aubry MC Vrtiska TJ Erlichman C Large cell carcinoma with calcitonin and vasoactive intestinal polypeptide-associated Verner-Morrison syndrome. Mayo Clinic Proceedings 2005 80 116–120. (10.1016/S0025-6196(1162968-6) 15667039

[21] Tsutsumi Y Immunohistochemical analysis of calcitonin and calcitonin gene-related peptide in human lung. Human Pathology 1989 20 896–902. (10.1016/0046-8177(8990103-2) 2550350

[22] Coombes RC Hillyard C Greenberg PB MacIntyre I Plasma-immunoreactive-calcitonin in patients with non-thyroid tumours. Lancet 1974 1 1080–1083. (10.1016/s0140-6736(7490557-1) 4135248

[23] Ellison M Woodhouse D Hillyard C Dowsett M Coombes RC Gilby ED Greenberg PB Neville AM Immunoreactive calcitonin production by human lung carcinoma cells in culture. British Journal of Cancer 1975 32 373–379. (10.1038/bjc.1975.237) 1233082 PMC2024744

[24] Hillyard CJ Coombes RC Greenberg PB Galante LS MacIntyre I Calcitonin in breast and lung cancer. Clinical Endocrinology 1976 5 1–8. (10.1111/j.1365-2265.1976.tb03797.x) 942890

[25] Silva OL Broder LE Doppman JL Snider RH Moore CF Cohen MH Becker KL Calcitonin as a marker for bronchogenic cancer: a prospective study. Cancer 1979 44 680–684. (10.1002/1097-0142(197908)44:2<680::aid-cncr2820440240>3.0.co;2-j) 476577

[26] Hansen M Hansen HH Hirsch FR Arends J Christensen JD Christensen JM Hummer L Kühl C Hormonal polypeptides and amine metabolites in small cell carcinoma of the lung, with special reference to stage and subtypes. Cancer 1980 45 1432–1437. (10.1002/1097-0142(19800315)45:6<1432::aid-cncr2820450622>3.0.co;2-z) 6244082

[27] Hansen M Rehfeld JF Stadil F Small cell carcinoma of the lung: relation of calcitonin to bone marrow metastases, parathormone and gastrin. Acta Medica Scandinavica 1979 206 215–218. (10.1111/j.0954-6820.1979.tb13497.x) 227233

[28] Hansen M Hammer M Hummer L Diagnostic and therapeutic implications of ectopic hormone production in small cell carcinoma of the lung. Thorax 1980 35 101–106. (10.1136/thx.35.2.101) 6246651 PMC471231

[29] Hansen M Hammer M Hummer L ACTH, ADH, and calcitonin concentrations as markers of response and relapse in small-cell carcinoma of the lung. Cancer 1980 46 2062–2067. (10.1002/1097-0142(19801101)46:9<2062::AID-CNCR2820460926>3.0.CO;2-X) 6253049

[30] Gropp C Havemann K Scheuer A Ectopic hormones in lung cancer patients at diagnosis and during therapy. Cancer 1980 46 347–354. (10.1002/1097-0142(19800715)46:2<347::aid-cncr2820460223>3.0.co;2-s) 6248192

[31] Coombes RC Dearnaley DP Ellison ML Neville AM Markers in breast and lung cancer. Annals of Clinical Biochemistry 1982 19 263–268. (10.1177/000456328201900415) 6127052

[32] Tabolli S Valtorta C Scarda A D'Erasmo E Minisola S Antonelli R Medori C Mazzuoli G Plasma calcitonin and tumors. Tumori 1983 69 227–230. (10.1177/030089168306900310) 6868140

[33] Roberts I Chopra S Warshaw AL Carcinoma of the lung with marked hyperamylasemia and elevated serum calcitonin. American Journal of Gastroenterology 1982 77 43–44. 6175207

[34] Sano T Saito H Yamasaki R Hamaguchi K Ooiwa K Shimoda T Hosoi E Saito S Hizawa K Immunoreactive somatostatin and calcitonin in pulmonary neuroendocrine tumor. Cancer 1986 57 64–68. (10.1002/1097-0142(19860101)57:1<64::aid-cncr2820570114>3.0.co;2-6) 2866833

[35] Eagle RC Jr Ehya H Shields JA Shields CL Choroidal metastasis as the initial manifestation of a pigmented neuroendocrine tumor. Archives of Ophthalmology 2000 118 841–845. (10.1001/archopht.118.6.841) 10865324

[36] Samuels T Cameron R Hirte H Osoba D Malkin DG Malkin A Imaging studies and the prognostic value of serum calcitonin in staging small-cell lung cancer. Tumour Biology 1987 8 211–217. (10.1159/000217524) 2834815

[37] Hansen M Hansen HH Tryding N Small cell carcinoma of the lung: serum calcitonin and serum histaminase (diamine oxidase) at basal levels and stimulated by pentagastrin. Acta Medica Scandinavica 1978 204 257–261. (10.1111/j.0954-6820.1978.tb08436.x) 211804

